# Silicon nanorod formation from powder feedstock through co-condensation in plasma flash evaporation and its feasibility for lithium-ion batteries

**DOI:** 10.1038/s41598-021-01984-y

**Published:** 2021-11-17

**Authors:** Akihiro Tanaka, Ryoshi Ohta, Masashi Dougakiuchi, Toshimi Tanaka, Akira Takeuchi, Kenichi Fukuda, Makoto Kambara

**Affiliations:** 1 Department of Materials Engineering, The University of Tokyo, Tokyo, 113-8656 Japan; 2 Shimane Institute for Industrial Technology, Matsue, Shimane Japan; 3 Takeuchi Electric Co. Ltd., Matsue, Shimane Japan

**Keywords:** Synthesis and processing, Batteries

## Abstract

Si nanowires/nanorods are known to enhance the cycle performance of the lithium-ion batteries. However, viable high throughput production of Si nanomaterials has not yet attained as it requires in general expensive gas source and low-rate and multiple-step approach. As one of the potential approaches, in this work, we report the fast-rate Si nanorod synthesis from low-cost powder source by the modified plasma flash evaporation and the fundamental principle of structural formation during gas co-condensation. In this process, while Si vapors are formed in high temperature plasma jet, molten copper droplets are produced separately at the low temperature region as catalysts for growth of silicon nanorods. Si rods with several micrometers long and a few hundred of nanometers in diameter were produced in a single process at rates up to 40 µm s^−1^. The growth of the Si nanorods from powder source is primarily characterized by the vapor–liquid–solid growth which is accelerated by the heat extraction at the growth point. The battery cells with the Si nanorods as the anode have shown that a higher capacity and better cyclability is achieved for the nanorods with higher aspect ratios.

## Introduction

Lithium ion batteries (LiBs) are light-weight and high-density storage that respond to the demand of the ever-increasing capacity in mobile electronics and electric vehicles. From the materials point of view, silicon is a promising candidate for anode of LiBs owing to its high theoretical capacity, which is 10 times higher than that of the conventional graphite^1^. However, it experiences a large volume change (up to 400%), associated with the formation of various Li–Si phases during repeated charge/discharge cycles. This causes cracking and fracture of the Si, which eventually leads to rapid capacity decay in a short cycles.

Structured materials can accommodate the huge expansion of Si and thereby retain its high capacity^2^. In particular, nanostructures, including Si nanoparticles^3–5^, nanowires^6–8^, and nanotubes^9,10^, have been used to suppress fracture owing to their small size and anisotropic structures, respectively. As for the nanowires, the pioneering work was carried out by Chan et al. in 2007^6^, who improved the initial coulombic efficiency by vertically aligning Si nanowires on stainless-steel substrate. Nguyen et al. further reported that the interconnecting structure among Si nanowires secures the attachment to the substrate, thereby improving the capacity retention^7^. The growth direction of Si nanowires is also known to influence the cyclability, because the degree of the volume expansion during lithiation is anisotropic and varies with crystal orientation. In situ TEM observation confirmed that the lithiated Si nanowires expand ~ 170% in the <110> direction, but less than 20% along the <111> direction^11^. Therefore, Si nanowires are designed to have a (111)-oriented structure, allowing the expansion only radially into the space between nanowires upon lithiation.

Si nanowires have been grown by a variety of processes, such as laser ablation, molecular beam epitaxy, SiO evaporation, solution-based methods and chemical vapor deposition (CVD)^12^. In particular, CVD is advantageous because it allows good control of the wire shape and high-yielding^1,13–17^. Despite the unique characteristics of each approach, vapor–liquid–solid (VLS)^18^ is a common and the key mechanism for the growth of Si nanowires. In VLS, Si vapor is introduced to a molten metal catalyst, and upon being supersaturated, Si grows as nanowires at the Si/metal catalyst interface^19^. Effective catalysts are elements that form a eutectic system with Si, such that Si is readily rejected by the molten catalyst and grows at temperatures slightly higher than the eutectic point. However, conventional VLS requires an expensive Si gas source, such as monosilane, in order for the molten catalyst and Si vapor to coexist at temperatures below the melting point of Si. Furthermore, because Si nanowires grow from a molten catalyst, a thin film of metallic catalyst has to be deposited on the substrate in advance and heated to form nanosized droplets just before VLS. This constraint means that VLS is a batch process. To be industrially feasible, Si nanowires are to be produced continuously from inexpensive raw materials.

Plasma flash evaporation (PFE) is used industrially for high throughput, continuous production of nanoparticles. In PFE, raw powders are fed into a plasma jet and undergo instantaneous evaporation and subsequent condensation, leading to homogeneous nucleation and growth of nanosized materials^3,20–24^. By adding various elements in powder form, alloy nanoparticles and/or composites nanoparticles can be produced depending on the altered co-condensation path of the high temperature vapors^20,21,23,25–28^. For example, nanocomposite Si–M particles, in which nanoparticles of a secondary element M are directly attached to Si nanoparticles, can be produced through heterogeneous nucleation during PFE. The LiB cells with these nanoparticles as the anode exhibit improved capacity retention owing to the reduced charge transfer resistance associated with the attached metallic nanoparticles^20,21,25^. As such, PFE is in principle capable of production of nanosized particulate materials from powder source. Moreover, it can be further configured to produce one dimensional structures, such as carbon nanotubes (CNTs)^29,30^. By injecting Ni based metal and carbon powders simultaneously, CNTs can be produced continuously from Ni nanoparticles that are thought to work as catalyst. However, there are no reports on formation of one dimensional Si materials using PFE to the best of our knowledge. One major reason for this is that, unlike carbon, silicon vapor is less stable at low temperature than the catalyst elements and tends to form a condensed silicide phase before the molten catalyst is created; that is, Si at the liquid phase coexists with the catalyst at the vapor phase, whereas VLS requires on the contrary the vapor-phase Si to contact the molten catalyst droplet.

In this work, we have carried out PFE by controlling the injection of Si and catalytic Cu powders separately to satisfy the VLS requirements and demonstrated for the first time the continuous growth of Si nanorods from powder source. The growth of the Si nanorods during co-condensation was quantitatively modeled and the strategies to control their structure are proposed. Furthermore, the feasibility of the Si nanorods for anodes of LiBs are discussed based on the charge–discharge cycling performance.

## Methods

### Nanorod formation with sPFE

**Figure 1:**
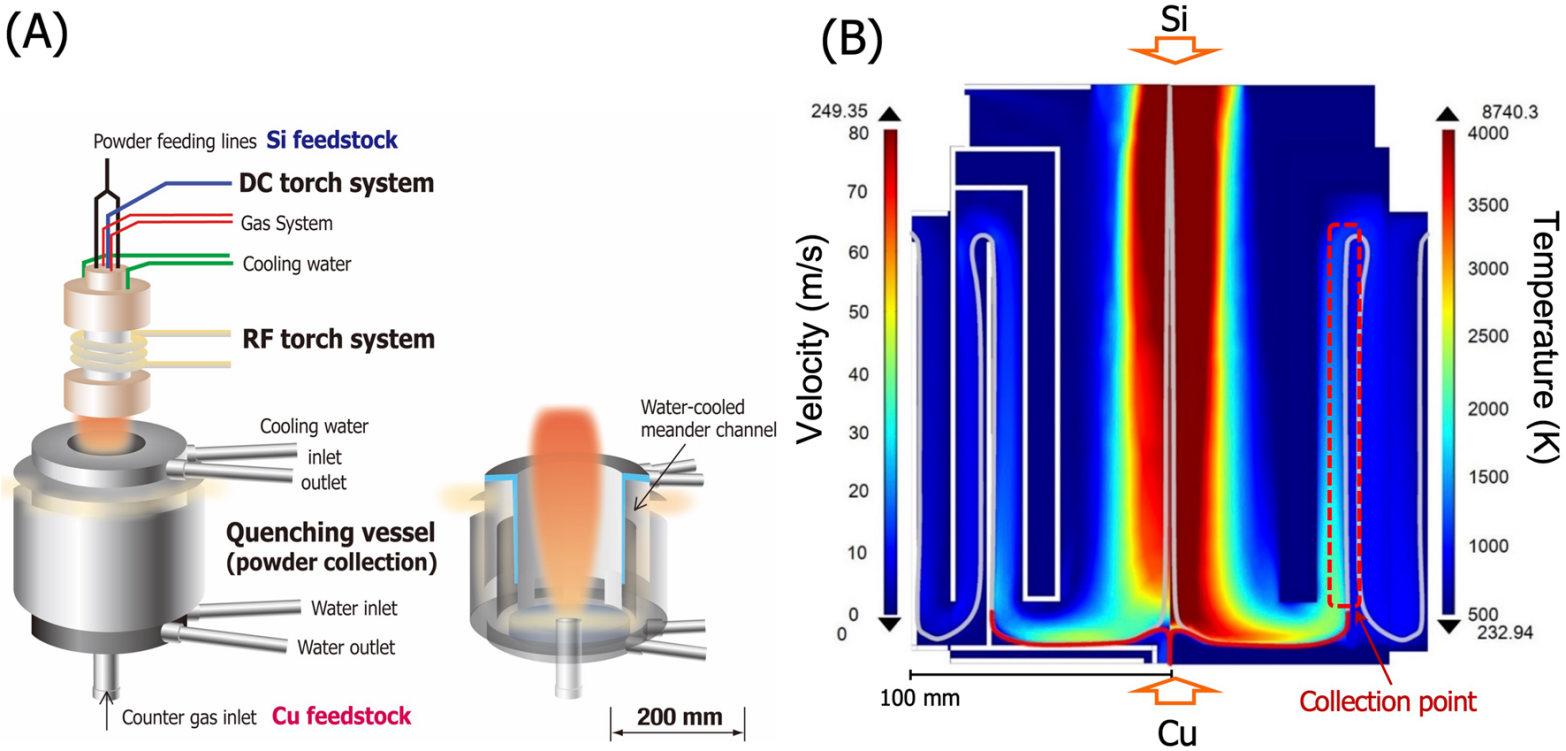
(**A**) Schematic illustration of the separate powder injection plasma flash evaporation (sPFE) apparatus. A hybrid plasma torch (DC and RF torches) is attached to the reaction chamber and a water-cooled gas quenching vessel is placed under the plasma torch. (**B**) Calculated plasma gas velocity and temperature distributions in the gas quenching vessel under the present sPFE condition. The detailed calculation condition can be found elsewhere^3^.

Si nanorods have been produced by separate powder injection plasma flash evaporation (sPFE) using a hybrid plasma spray system (VT-HB, JEOL Co.). Inductively-coupled plasma (ICP) was generated with Ar and H_2_ at an radio-frequency (RF) input power of 90 kW and a 3-kW direct current (DC) jet was superimposed axially from the top of the ICP to stabilize the plasma flame upon powder injection at high rates. A water-cooled gas-quenching vessel was placed under the plasma torch such that the high-temperature vapors condensed immediately; this also ensured that the resulting nanomaterials were attached on the vessel wall and collected afterwards. Metallurgical-grade Si powder with a mean diameter of 16 µm (purity: 99.9%, Shinetsu Co. Ltd.) was used as the Si vapor source and Cu powders with a mean diameter of 5 µm (purity: 99.9%, Kojundo-Chem. Co.) was used as the catalyst feedstock. As shown in Fig. 1A, the Si powder was introduced axially from top to the highest-temperature zone of the plasma jet, while the catalyst Cu powder was separately injected upward from the bottom of the quenching vessel to a low-temperature zone of the plasma tail flame. Under the optimal plasma conditions, our sPFE allows us to completely vaporize Si powders and completely melt the Cu powders. This satisfies the VLS requirement, “coexistence of Si vapor and Cu catalyst droplets”, within the collection vessel. The Si and Cu raw powders were introduced by a powder feeder (TP-99010FDR, JEOL Ltd.) at a constant rate to maintain the target Si:Cu ratio in the plasma. To identify the effect of the catalytic Cu powders on the shape of Si nanorods, different powder feeding rates for Si and Cu were selected as listed in Table 1. The detailed parameters used in the sPFE are listed in Supplementary Table S1.

**Table 1:** Parameters used in the separate injection plasma flash evaporation experiment.

Condition	Feeding rate (g min^−1^)	
	Si	Cu
I	1	1
II	1	2
III	2	2

Powder feeding rate of Si and Cu feedstocks.

### Particle characterization

Morphological observation of the nanorods was carried out using field emission scanning electron microscopy (FESEM, H-800, Hitachi Co.) with energy dispersive X-ray spectrometry (EDS), and using high resolution transmission electron microscopy (HRTEM, H-9500, Hitachi-Hitech Co.). The phases after sPFE were identified by X-ray diffraction (XRD, D2 PHASER, Bruker Co.) and Rietveld analysis using TOPAS 4 software (Bruker Co.).

### Battery assembly and electrochemical analysis

After being screened with a 45-µm mesh sieve, the plasma processed materials were mixed with Super-P carbon conducting reagents and polyimide binder. The resultant slurry was applied onto a Cu foil current collector and dried to a thickness of approximately 10 µm, excluding the foil. The anodes were cut in a circle and assembled in a 2016 half cell together with lithium metal (Honjo Metal Co.) as the counter electrode and 1 M of lithium hexafluorophosphate (LiPF_6_) dissolved in a solution of 5:1:1 (v/v) ethylene carbonate, diethyl carbonate and fluoroethylene carbonate as the electrolyte. The cells containing nanorods formed under different sPFE conditions were identically assembled. The battery performance was analyzed using an ACD-M01A (Asuka Co.) cycle test device at a constant current of 0.1 mA (corresponding to a rate of 0.02C) for the first three cycles and at 0.5 mA (0.1C rate) for the remaining cycles with a cut-off voltage of 0–1.5 V at a controlled temperature of 23 °C. Symmetric cells with the sPFE-produced nanorods as anodes for both electrodes were assembled and the impedance was measured using electrochemical impedance spectroscopy (EIS, Bio-Logic Co.).

## Results and discussion

### Structural characteristics of the Si nanorods

**Figure 2:**
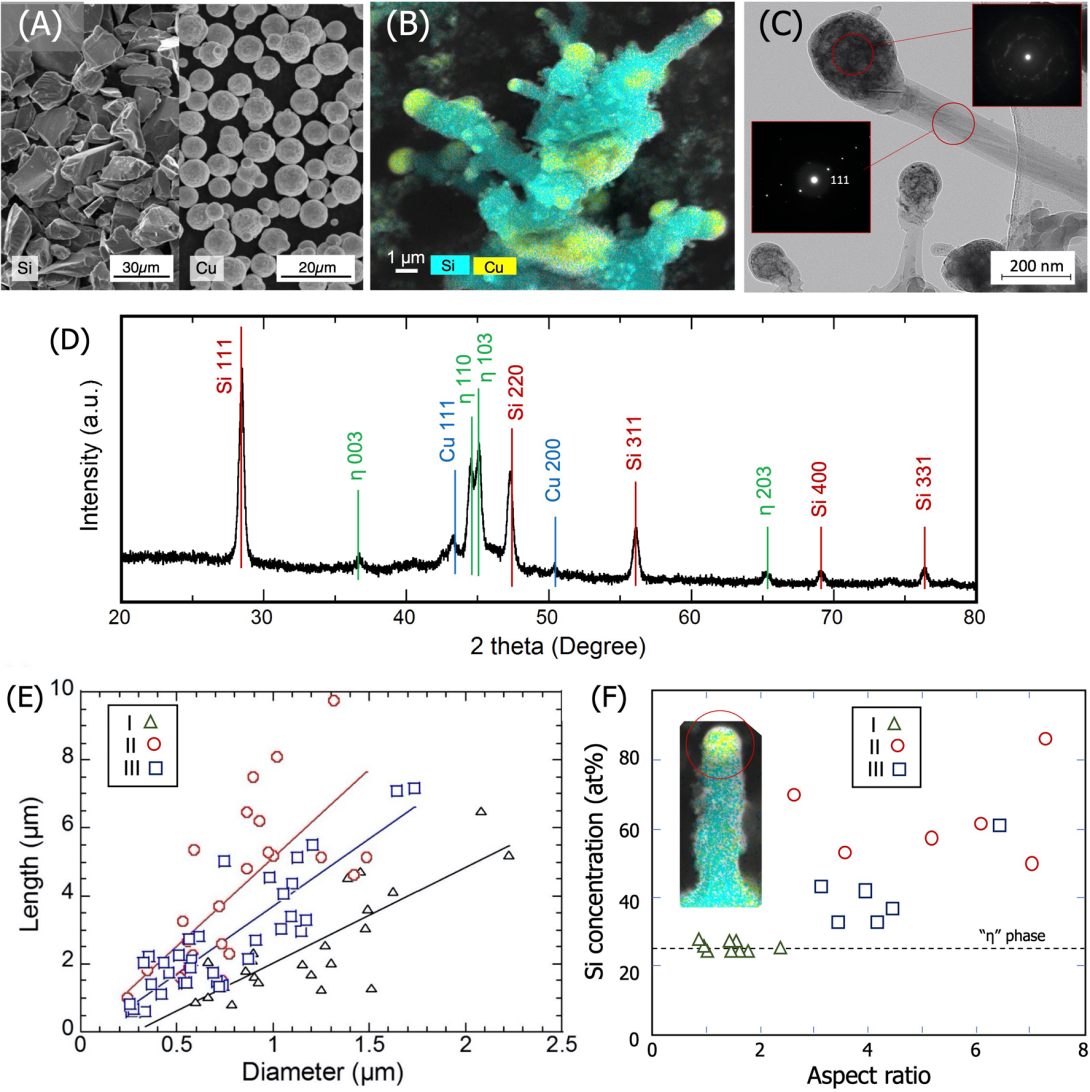
Scanning electron microscope (SEM) images of (**A**) Si and Cu powders, (**B**) Si nanorods (condition II) with elemental mapping analyzed by energy-dispersive X-ray spectroscopy (EDS). (**C**) High-resolution transmission electron microscopy right-field image of the Si nanorods (condition II) and electron diffraction patterns at the rod and the cap. (**D**) X-ray diffraction pattern of the nanorods (condition II). (**E**) Length and diameter of the nanorods observed in the SEM images. (**F**) Si concentrations in the hemispherical cap measured using SEM–EDS.

Figure 2A shows the SEM image of the Si and Cu powders used for sPFE. The Si powder has a crushed form and the Cu has a globular shape. These morphologies are characteristic of the preparation methods of mechanical milling and gas atomization, respectively. After sPFE, Si becomes rod shaped and Cu forms a small hemispherical cap on the tip of each rod, as seen in Fig. 2B. The diameter and length of the Si rods vary, but the average size is smaller than that of the initial powders for all conditions. SEM images for other conditions are shown in Supplementary Fig. S1. Therefore, the Si powder is considered to vaporize and condense subsequently to form rod-shape structure. It is also noted that Cu and Si nanoparticles are attached to the sides of the rods, which is evidence of continuous supply of Si and Cu during the growth of Si nanorods.

The HRTEM image in Fig. 2C shows the rod-shaped materials at a smaller length scale. The electron diffraction pattern (EDP) at the rod region shows an unique alignment of the diffraction spots from the Si(111) in one direction, which confirms the unidirectional growth of Si in the <111> direction. By contrast, in the EDP of the tip region, many faint diffraction spots are observed to form rings, which indicates that fine crystalline particles are formed in random orientations. This is further supported by the presence of the dark-contrasted particles in the cap in the bright-field image. Considering the EDS analysis and the length of the diffraction spot of EDP, it is concluded that these fine nanoparticles are the Cu_3_Si “h” phase alloy.

Figure 2D shows the XRD pattern of the materials processed under condition I. The XRD pattern is basically similar for the other conditions. This XRD pattern confirms the presence of the mixture of crystalline Si, Cu and the h phase in the sPFE product. However, the peak intensity of Cu is smaller than that of the h phase and no other Si–Cu alloy phase is detected. Therefore, most of the injected Cu is considered to alloy with Si and form the h phase as a spherical cap at the rod tip. The relative amounts of the existing phases are listed in Supplementary Table S2 in the Supplementary Information.

The diameter and length of the rod-shaped materials measured from the SEM images are plotted in Fig. 2E. The size of the material depends on the growth conditions, ranging from nanorods with diameters of several hundred nanometers to large rods ~ 10 µm in length and 1.5 µm in diameter. Even so, there observed a linear correlation between length and diameter. The aspect ratio (AR) of the rod, represented by the slope of the linear relationship, varies from 1 under condition I to 8 under condition II. According to the definition^1^, nanomaterials produced by sPFE under the present condition is categorized as nanorod because AR is smaller than 20.

The Si concentration in the Cu-rich hemispherical cap measured by SEM–EDS is also plotted in Fig. 2F as a function of the aspect ratio. For all three conditions, the minimum Si concentration in the cap is 25%; this increases slightly with aspect ratio. The Si concentration of the Cu_3_Si phase is 25% and is 32% at the eutectic point. This, therefore, confirms that the cap is composed of a mixture of Cu_3_Si and Si.

### Limiting step of Si nanorod growth

For VLS growth in a bottom–up approach, Si grows having the spherical Cu catalyst at the tip of the wire. During growth, while gas phase Si is supplied to one side of the catalytic droplet, Si is supersaturated and rejected by the droplet unidirectionally on the other side. Upon cooling, the Si-saturated catalyst droplet then solidifies to form Cu_3_Si via a eutectic reaction. The formation of Cu_3_Si at the cap of the rod is therefore the evidence of the directional growth of the rod from the Cu-rich hemispherical cap. Thus, the results above confirms that VLS growth occurs in sPFE. Furthermore, Si nanorod grows in the <111> direction is (based in the EDP results) for the rods with diameter greater than 100 nm. This agrees with a previous report that the growth orientation depends on the rod diameter, whereby the <110> direction is preferred for rods smaller than 20–30 nm and the <111> direction is preferred for larger rods^16^. This further supports the interpretation that nanorods produced from powders by sPFE are formed via the VLS mechanism fundamentally.

The Si concentration in the catalyst droplets is high especially for the rods with high aspect ratio (Fig. 2F). Considering that growth occurs via the VLS mechanism, we attribute this Si accumulation in the catalyst droplet to the Si consumption rate for growth being slower than the Si supply from the vapor phase. Moreover, this indicates that Si nanorod growth is mainly controlled by the crystallization step at the catalyst/rod interface, not by the incorporation step (gas phase Si supply) or by the Si diffusion in the molten catalyst^31^. Although the longer Si nanorods are expected to form under condition III, at which the Si powder feeding rate is higher than the other two conditions, the length is similar to or smaller than that in condition II. Since the growth temperature is below the melting point of Si, especially when Si flux is large at high feed rate, Si is likely to solidify and cover the catalyst rather than being dissolved in the droplet, which could retard the Si nanorod growth accordingly.

Si nanorod production was completed within 10 min. If the nanorods grow via the VLS mode at the reported growth velocity (~ 1 µm s^−1^)^32^, a length of ~ 600 µm would be expected. However, we obtained nanorods up to 10 µm in length. Because the gas quenching vessel is water-cooled, the nanomaterials produced inside are expected to cool immediately upon attachment to the vessel wall. Thus, the growth period will be primarily controlled by the cooling rate on the wall. In fact, for a constant Si powder feeding rate, when the powder feeding rate of the Cu catalyst is increased (from condition I to II), the average aspect ratio increases. This highlights that the addition of Cu modifies the SiNR structure and influences the heat extraction towards the vessel wall.

In principle, to grow Si nanorods, Si needs to be supersaturated in the molten Cu catalyst. To attain supersaturation, the catalyst temperature has to be in the Si(s) + L two-phase field of the Si–Cu binary phase diagram (Supplementary Fig. S2), on the condition that Si is supplied to the Cu catalyst via the Si vapor all the time during processing. However, according to the plasma gas-flow simulation shown in Fig. 1B, the gas temperature from the inlet to the collection point is higher than the Si melting point. This indicates that Si is less likely to be crystallized during the flight of Cu droplets, and also that growth starts only after Cu attachment on the vessel wall. The heat transfer to the vessel wall is therefore the primary limiting step of the growth of Si nanorods.

The primary growth path of Si nanorods during sPFE is thus summarized in the following stages:

1. Si powders vaporize while Cu powders are melted separately.
2. Once Si vapor and molten Cu droplet meet together, Si starts to dissolve in the Cu droplet during flight.
3. When the droplets arrive at the collection point, Si starts to grow at a rate that depends on the cooling speed, if enough Si amount is supplied during flight. Growth in the stage can be described as directional solidification (DS) mode.
4. Si is supplied continuously to the catalyst during nanorod growth. This is therefore recognized as the quasi steady-state growth and can be described as the VLS growth mode.
5. When the Cu particle temperature falls below the eutectic temperature, nanorod growth stops and the molten catalyst also solidifies.

### Formation of catalytic Cu droplets

As shown in Fig. 1B, the gas temperature at the inlet of the vessel is higher than 4000 K and the gas is cooled to 2500 K near the collection region. The homogeneous nucleation temperature of Si under the similar condition is calculated to be 2200 K^23^. Therefore, the Si raw powders should be vaporized in the torch and Si vapor is conveyed to the collection point. While, the counter flow gas from the bottom of the vessel immediately meets the down-flow gas and gas temperature increases rapidly up to 3000 K. Once they merge, the gas is cooled together along the same gas stream line with the down-flow. Assuming that Cu particles are transferred in the upward counter gas flow, the particle heating history is calculated. For Cu particles with an average diameter ($d_{{\text{p}}}$) of 5 µm, using the Cu thermal conductivity and the heat transfer coefficient, $h$, between the particle and gas, the Biot number is estimated to be 0.02. Therefore, Newtonian heating could be assumed and the lumped capacitance model is applied for Cu heating. The temperature evolution of Cu particles is then obtained by solving the following energy balance equations. The total heat flux on the Cu surface $\dot{Q}_{{\text{p}}}$ is described by the heat transfer to or from the plasma and the radiation loss as Eq. (1).

1 $$\pi d_{{\text{p}}}^{2} h\left( {T_{{\text{s}}} - T_{{\text{p}}} } \right) - \varepsilon \sigma_{{{\text{SB}}}} \left( {T_{{\text{p}}}^{4} - T_{{\text{w}}}^{4} } \right) = \dot{Q}_{{\text{p}}}$$

Here, $\sigma_{SB}$ is the Stephan–Boltzmann constant and the subscript p, s, and w denote particle, particle surface, and the vessel wall, respectively. Under the condition of our experiment, the Nusselt number $Nu_{{\text{f}}}$ is expressed as Eq. (2)^33^,

2 $$Nu_{{\text{f}}} = \left( {2 + 0.6Re_{{\text{f}}}^{1/2} Pr_{{\text{f}}}^{1/3} } \right)\left( {\frac{{\rho_{{\text{f}}} \eta_{{\text{f}}} }}{{\rho_{{\text{p}}} \eta_{{\text{p}}} }}} \right)^{0.6} \left( {\frac{{c_{{{\text{p}},{\text{f}}}} }}{{c_{{{\text{p}},{\text{p}}}} }}} \right)^{0.38} ,$$

and the heat transfer coefficient is given by,

3 $$h = \frac{{\lambda_{{\text{f}}} }}{{d_{{\text{p}}} }}Nu_{{\text{f}}} f_{3} ,$$

where $Re_{{\text{f}}}$ is the Reynolds number and $Pr_{{\text{f}}}$ is the Prandtl number of the gas. $\rho$, $\eta$ and $c_{{\text{p}}}$ are the density, viscosity, and specific heat, respectively, and the subscript ${\text{f}}$ represents the gas. $f_{3}$ is the non-continuum parameter that changes with Knudsen number. Under the present condition, $f_{3}$ is assumed to be 0.1. For the lumped capacitance model, $\dot{Q}_{{\text{p}}}$ during heating and during phase changes, such as melting and vaporization, is expressed by Eq. (4):

4 $$\dot{Q}_{{\text{p}}} = \left\{ {\begin{array}{*{20}l} {\frac{\pi }{6}d_{{\text{p}}}^{3} \rho_{{\text{p}}} c_{{\text{p}}} \frac{{{\text{d}}T_{{\text{p}}} }}{{{\text{d}}t}}} \hfill \\ {\frac{\pi }{6}d_{{\text{p}}}^{3} \frac{{{\text{d}}H_{{\text{p}}} }}{{{\text{d}}t}}} \hfill \\ { - \frac{\pi }{2}d_{{\text{p}}}^{2} H_{{\text{b}}} \frac{{{\text{d}}d_{{\text{p}}} }}{{{\text{d}}t}}} \hfill \\ \end{array} } \right.$$

Here, ${\text{d}}H_{{\text{p}}} /{\text{d}}t$ represents the change in the enthalpy. The time integration of $H_{{\text{p}}}$ needs to exceed the latent heat of fusion $H_{{\text{m}}}$ at the melting point for complete melting. Furthermore, $H_{{\text{b}}}$ is the latent heat of vaporization and the third equation of Eq. (4) expresses the change in the enthalpy due to the decrease in the size of droplet during vaporization at the boiling point.

Although Si and Cu are injected separately in sPFE, Si vapor and Cu droplets meet together and Si dissolves in the Cu droplet during flight. The amount of the Si dissolved in the Cu droplet is thus given by the time integration of the Si vapor flux arriving at the droplet surface. The Si flux, $\Gamma$, is expressed by the Hertz–Knudsen equation,

5 $$\Gamma = \frac{{p_{{{\text{Si}}}} }}{{\sqrt {2\pi mkT_{{\text{p}}} } }} ,$$

where $p_{{{\text{Si}}}}$ is the partial pressure of Si. For a certain infinitesimal time duration, ${\text{d}}t$, a change in the Si concentration in a Cu–Si droplet, ${\text{d}}C_{{\text{d}}}$, is thus obtained by the following equation using the droplet surface area, $S_{{\text{p}}}$, which is a function of the time-dependent droplet size, the total number of moles, $N$, and number of moles of Si, $n_{{{\text{Si}}}}$, in the droplet:

6 $${\text{d}}C_{{\text{d}}} = \frac{{\Gamma S_{{\text{p}}} \left( {d_{{\text{p}}} } \right){\text{d}}t + n_{{{\text{Si}}}} }}{{N + \Gamma S_{{\text{p}}} \left( {d_{{\text{p}}} } \right){\text{d}}t}}$$

Considering that the density of the molten Cu–Si varies with Si concentration^34^, $C_{{\text{d}}}$ is obtained by integrating Eq. (6) with respect to time.

**Figure 3:**
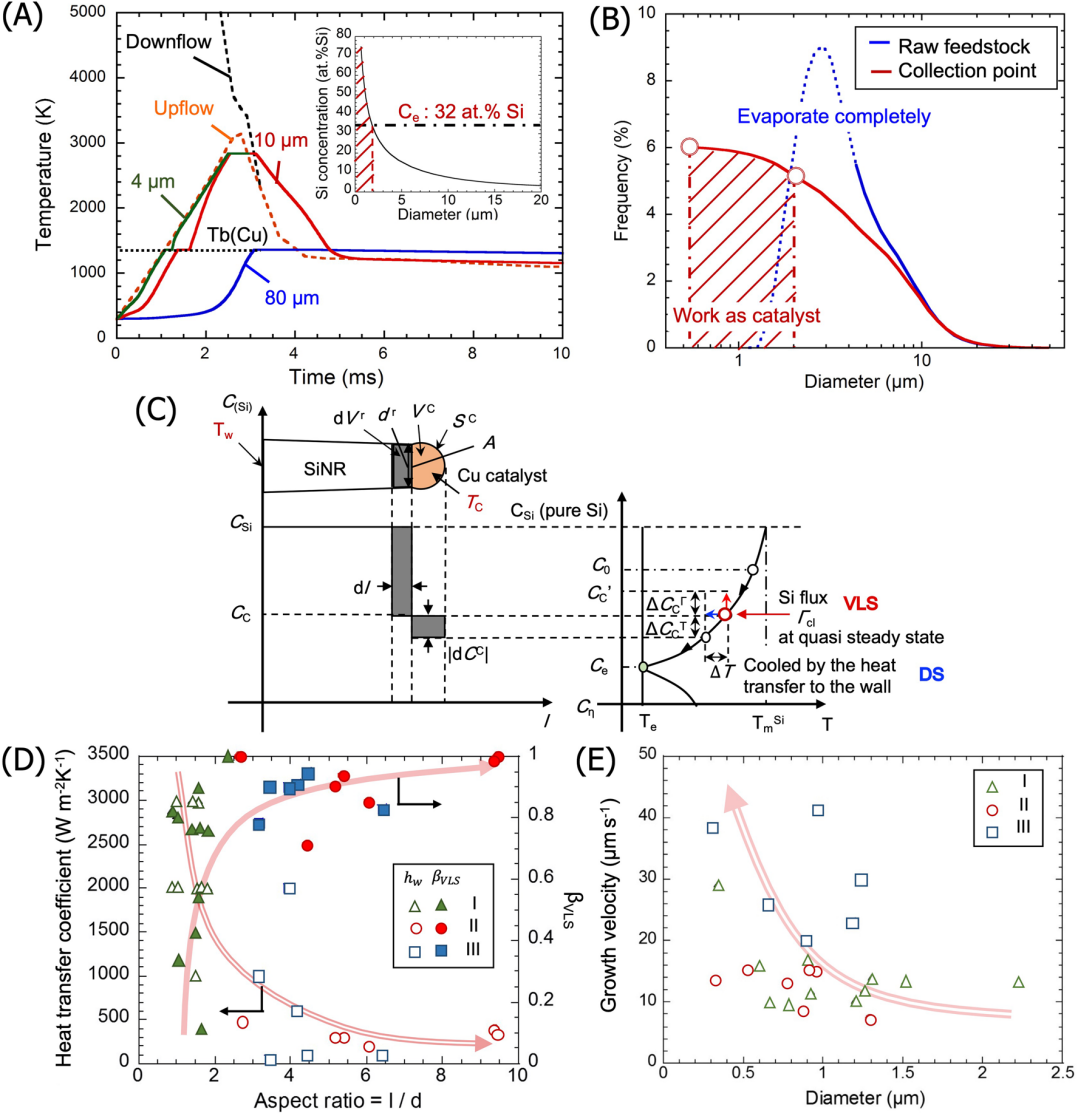
(**A**) Heating histories of Cu particles with different initial diameters. The inset is the Si concentration in the Cu droplets at the collection point. (**B**) Size distribution of the initial Cu particles and of the molten catalyst for DS mode. (**C**) Schematic representation of the mass transfer during nanorod growth, showing the Si redistribution at the growth interface (left) and the corresponding concentration in the Si–Cu binary phase diagram (right). (**D**) Relationship between the aspect ratio of the nanorods and the heat transfer coefficient and the VLS contribution. (**E**) The total growth velocity of nanorods as a function of rod diameter.

The merger of down- and upflow gas streamlines and their passage through the collection point are shown in Fig. 3A. This figure also compares the thermal histories of the Cu particles with different size. Cu particles with diameters smaller than 4 µm are seen to reach the boiling point and vaporize completely in a few milliseconds. By contrast, the large Cu particle with an 80-µm diameter reaches the melting point but remains at this temperature, suggesting that this particle does not melt completely. According to the calculation, Cu particles with the initial diameters from 4.2 to 75 µm attain a fully-molten state without complete vaporization.

The inset of Fig. 3A shows the Si concentration ($C_{{\text{d}}}$) in the Cu droplets that arrive at the collection point as a function of the droplet size. For Si nanorod growth upon arrival at the collection position, $C_{{\text{d}}}$ must be at least greater than the Si concentration at the eutectic point (i.e. > 32 at%) to gain supersaturation. Cu droplets that satisfy this requirement are approximately 2 µm or smaller. In fact, in the SEM image of Fig. 2B, the spherical caps at the Si nanorod tip are observed to be in the range of 0.2–2 µm. Cu droplets with 2-μm diameter are obtained from Cu particles with an initial size of 4.5 μm after partial vaporization of Cu during flight. Therefore, as Fig. 3B compares the size distribution of the Cu droplets at the collection point with that of the initial particles, only initial Cu particles with a size between 4.2 and 4.5 µm can melt completely and satisfy the saturation condition of $C_{{\text{d}}}$ > 32 at% at the collection point.

In principle, there are possibilities that Cu particles with other sizes become catalysts: Large initial particle with 4.5–75 µm can also work as catalysts if enough Si is supplied after arriving at the collection point. However, no Si rods having a cap with 2-µm or larger are observed in the SEM. This suggests in turn that the heat extraction on the vessel wall is relatively fast to solidify quickly, comparing to the Si vapor supply to saturate such large particles. For particles smaller than 4 μm, in contrast, although they vaporize completely during flight, they can become catalysts if Cu and Si vapors are mixed well and condense to attain a molten state with $C_{{\text{d}}}$ > 32 at%. Small Si nanorods observed in the TEM image may have resulted from the latter formation path.

### Characteristics of Si nanorod growth in sPFE

Si nanorods grow from the Si dissolved Cu droplets once the droplets become supersaturated at the wall upon cooling. Therefore, the mass transfer in nanorod growth can be described by the conventional directional solidification. Because the Si concentration in a small Cu droplets can be assumed to be homogeneous, the redistribution of Si at the growth interface is described by the lever-rule mass balance, as schematically shown in Fig. 3C and expressed by Eq. (7),

7 $$\left( {c_{{{\text{Si}}}} - c_{{\text{c}}} } \right){\text{d}}V_{{\text{r}}} = - V_{{\text{c}}} {\text{d}}c_{{\text{c}}} ,$$

where the rod growth volume, ${\text{d}}V_{{\text{r}}}$, is the product of the catalyst/rod interfacial area, $A_{{\text{r}}} \left( {c_{{\text{c}}} } \right)$, and the rod growth length of the rod, ${\text{d}}l$. The subscripts ${\text{c}}$ and ${\text{r}}$ represent the cap and the rod of Si nanorod, respectively. $A_{{\text{r}}}$ is determined from the Neumann triangle relationship at the catalyst-rod junction^35^, using the Si/Cu contact angle of 130˚ observed in the SEM image. Moreover, $A_{{\text{r}}} \left( {c_{{\text{c}}} } \right)$ is introduced as a function of $c_{{\text{c}}}$ because the Si–Cu density increases with increasing Si concentration^34^. $V_{c}$ is the volume of the catalyst, which is expressed as a function of $d_{{\text{r}}}$.

Meanwhile, because of the small size of the Cu–Si droplets and Si nanorods, the heat extraction can be assumed to be controlled by the heat transfer at the interface between the Si nanorod and the wall. By introducing the heat transfer coefficient, $h_{{\text{w}}}$, the change in the droplet temperature is expressed based on the heat flux conservation,

8 $$\frac{{{\text{d}}T_{c} }}{{{\text{d}}t}} = \frac{{A_{{\text{r}}} h_{{\text{w}}} \left( {T_{{\text{w}}} - T_{{\text{c}}} } \right)}}{{\rho_{{\text{c}}} \left( {c_{{\text{c}}} } \right)V_{{\text{c}}} c_{{\text{p}}}^{{\text{c}}} }} .$$

Here, $c_{{\text{p}}}^{{\text{c}}}$ is the specific heat capacity of the Cu droplets, and $T_{{\text{c}}}$ and $T_{{\text{w}}}$ are the cap and the vessel wall temperature, respectively. Using Eqs. (7) and (8), the rod length, $l$, is thus expressed as a function of $c_{{\text{c}}}$, which is determined by the time-dependent $T_{{\text{c}}}$ along the liquidus line under the local equilibrium assumption,

9 $$\frac{{{\text{d}}l}}{{{\text{d}}c_{{\text{c}}} }} = - \frac{{V_{{\text{c}}} }}{{A_{{\text{r}}} \left( {1 - c_{{\text{c}}} \left( {T_{c} } \right)} \right)}} .$$

In addition to the Si supply during flight, Si is supplied to Cu during nanorod growth on the vessel wall; that is, the VLS mode should proceed along with the DS mode. The total amount of Si supplied to the catalyst is thus estimated experimentally from the volume of Si nanorods and the Si concentration remaining in the Cu cap. Considering the constant Si flux given by Eq. (5), the total growth time is calculated, as shown in Supplementary Fig. S3. The growth time increases with increasing the nanorod length, reaching a maximum value of approximately 800 ms. The Si amount supplied during flight (DS mode) is also obtained from Eq. (6), and the corresponding growth time is calculated to be 3.85 ms, which is much smaller than the total growth time. This difference suggests that the remaining growth time for the VLS mode controls the nanorod growth largely. The contribution of the VLS mode to the overall growth is thus quantified by setting the factor $\beta_{{{\text{VLS}}}}$, which is the relative amount of Si for the VLS divided by the total Si in the rod, as shown in Fig. 3D. Moreover, for a given Si flux, the heat transfer coefficient at the vessel wall and the corresponding growth velocity are estimated for each nanorod. These growth characteristics are plotted in Fig. 3D and E. It is noted that because the gas temperature at the collection point is below the Si homogeneous nucleation temperature^3^, the effective Si flux during VLS is modified accordingly by taking into account the Si supply in the form of nanoclusters.

It is seen from Fig. 3D that when Si nanorods with large aspect ratios are obtained, the contribution of VLS becomes large while the corresponding heat transfer coefficient becomes small. This result indicates that the nanorods grow longer if the heat extraction is not significant and thus the growth time becomes long. In particular, for condition II and III, when more Cu powders are injected, the heat extraction capability seems to decrease. Although the addition of metallic Cu is expected to increase the overall heat transfer, Cu appears to retain the heat from the plasma by forming branching structures of nanorods, as observed in Fig. 2B. Furthermore, Fig. 3E shows a general tendency of the velocity increasing with decreasing the rod diameter. For a Cu catalyst, similar size dependency is reported for larger rods with a radius greater than 2 µm^32^, whereby the growth velocity increases with decreasing the growth temperature, reaching 1.5 µm s^−1^ at 1300 K. The average growth velocity of Si nanorod in sPFE is one order of magnitude faster than this reported velocity. In contrast with the steady-state growth in the conventional VLS, the nanorod growth in sPFE is primarily limited by rapid cooling. Therefore, the diffusion and incorporation of Si at the growth interface are plausibly accelerated by the forced heat extraction. Moreover, considering the feed rate of the powder and the yield of catalytic Cu droplet formation, the processing speed of Si nanorod is estimated to be 8.27 g h^−1^ for condition II, which is higher than the reported yield of 45 mg h^−1^ for solution based processing^36^.

### Electrochemical properties of cells with Si nanorods as anode

**Figure 4:**
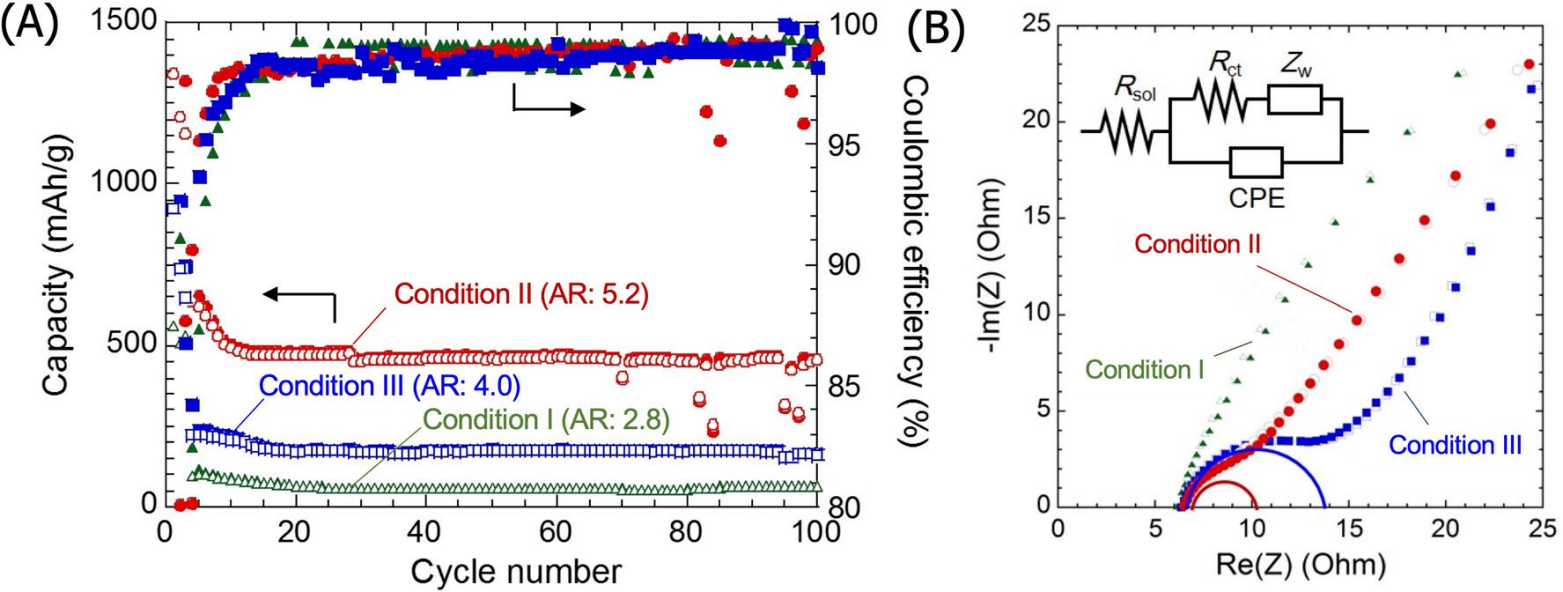
(**A**) Capacity and coulombic efficiency as a function of cycle number for cells prepared with Si nanorods of differing aspect ratio as the anode material. (**B**) Cole–cole plots for symmetric cells with different Si nanorods anode.

Figure 4A shows the charge–discharge capacity and the coulombic efficiency of the cells using anodes composed of the Si nanorods. As a general tendency, the initial charge capacity reaches nearly the theoretical capacity that is expected from the Si phase amount in the nanorods (Supplementary Table S2). No appreciable contribution of silicides is observed. However, it decreases sharply during the first few cycles. Nevertheless, a capacity is maintained with relatively good cyclability for at least 100 cycles, in contrast with the gradual capacity decay observed for the cells with nanoparticles produced by PFE^3^. This behavior is similarly observed for cells containing nanorods formed under all three conditions. However, the Si nanorods with the highest aspect ratio (condition II) is seen to attain the highest specific capacity. The change in differential capacity upon cycling (Supplementary Fig. S4) was fundamentally similar among all conditions, except the intensity, with the peak positions appearing at the same potential and profiles being identical between 50 and 100th cycle. This behavior also underlines the good cyclability of these cells.

Since the average diameter of Si nanorod in the present work is relatively larger than the critical size^1^, the nanorods partly fractured, which may explain the initial capacity drop. Nevertheless, as anode materials, 1D Si nanorods grown in the <111> direction results in a better cyclability than the isotropic Si particles; the improvement becomes pronounced as the rod length increases. This tendency likely relates to the electronic/ionic conducting paths being maintained with high-aspect-ratio Si nanorods, as confirmed by the EIS measurement shown in Fig. 4B. The Nyquist plots of symmetrical cells with different Si nanorods look different, but the equivalent circuits that fit the spectra are similar (inset of Fig. 4B). As the estimated values of the resistive elements are listed in Supplementary Table S3, the semicircle diameter that is associated with the charge transfer resistance, *R*_ct_, is reduced significantly with increasing the aspect ratios. There is no tendency between *R*_ct_ and the phase amount of the electrical conducting Cu and Cu silicides in the anode, indicating that the interface between Si and Cu catalyst is not electrochemically resistive. Therefore, as the cells were assembled identically, we attribute the enhanced charge transfer for longer nanorods to the continuing electric-conducting network in the anode.

## Conclusion

We have demonstrated silicon nanorods formation from a powder feedstock using separate-injection plasma flash evaporation. The growth of the nanorods during sPFE is fundamentally characterized by the conventional VLS, and the forced heat extraction assists to increase the average growth velocity.

The nanorod diameters range from 50 nm to 2 µm, which are slightly larger than the critical size for fracture of silicon nanowires during Li-ion battery operation. As a result, although the initial high capacity decreases significantly at the initial charge–discharge cycles, the one-dimensional structure with aspect ratios as high as 10 favorably decreases the charge transfer resistance, thereby improving the cyclability of the cells. The growth model reveals that the diameter of the Si nanorod is determined by the size of the Cu droplet and the length is controlled by the cooling during the growth. Therefore, it is anticipated to produce longer Si nanowires with smaller diameter, optimal for battery anodes, by controlling the temperature at the growth point to be high to maintain small Cu particle as active molten catalyst.

## Supplementary Information

Supplementary Information.
