# Supplementary material for: Silicon nanorod formation from powder feedstock through co-condensation in plasma flash evaporation and its feasibility for lithium-ion batteries
Source: Sci Rep. 2021 Nov 17;11:22445. doi: 10.1038/s41598-021-01984-y (PMC8599667; doi:10.1038/s41598-021-01984-y)
Supplement: Supplementary file 1 — Supplementary Information. [file 41598_2021_1984_MOESM1_ESM.pdf]

Supplementary Information

Silicon nanorod growth from powder feedstock through co-condensation in plasma flash evaporation and its feasibility for lithium-ion batteries

Akihiro Tanaka[1], Ryoshi Ohta[1], Masashi Dogakiuchi[2], Toshimi Tanaka[3], Akira Takeuchi[3], Kenichi Fukuda[2] & Makoto Kambara[1*]

Table S1. Parameters used in the present sPFE.

Parameter	
RF input power [kW]	90
Plasma gas Ar flow rate (radial) [slm]	140
(tangential) [slm]	30
Plasma gas H_2 flow rate (radial) [slm]	30
Process pressure [Torr]	400
Base pressure [Torr]	0.15
Process time [min]	10
DC input power [kW]	3
DC Ar gas [slm]	10
Powder carrier Ar gas flow rate [slm]	3.5
Counter Ar gas flow rate [slm]	50

Table S2. The relative amount of the existing phases estimated by XRD-Rietveld analysis.

condition	Si (at.%)	Cu (at.%)	$Cu_3Si(\eta)$ (at.%)
I	74.2	8.28	17.6
II	81.8	3.87	14.3
III	90.5	2.78	6.67

Table S3. The relative amount of the existing phases estimated by XRD-Rietveld analysis.

	I	II	III
R_{sol} (Ω)	6.169	6.630	6.457
R_{ct} (Ω)	56.93	2.134	7.132
Z_w ($\Omega\ s^{-1}$)	3.447	752.3	242.5
T_{CPE} ($\mu F\ s^{p-1}$)	5.735	0.7511	5.597
p	0.8129	1.000	0.8727

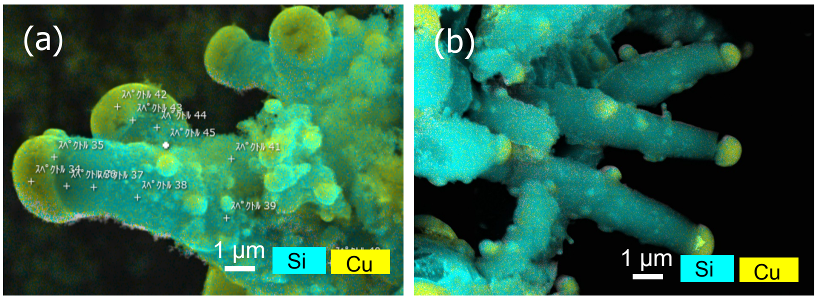

Figure S1. Typical SEM images of the processed materials under condition I (a) and III (b).

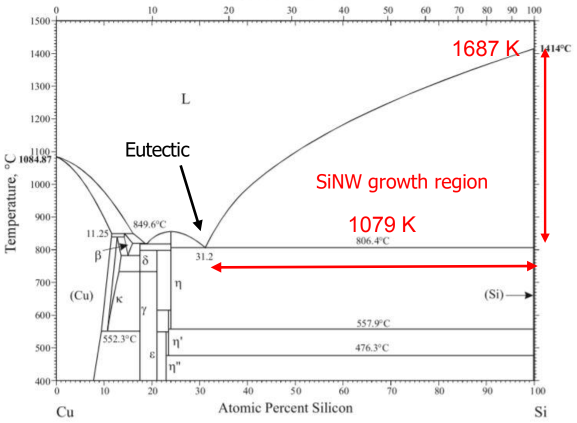

Figure S2. Si-Cu binary phase diagram showing the region for SiNR growth.

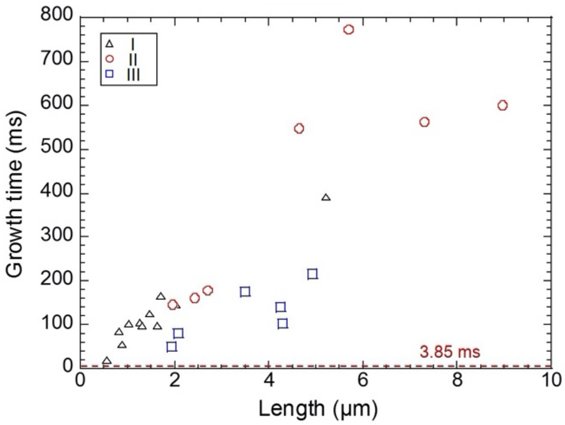

Figure S3. Total growth time estimated by the experimentally observed SiNR and the model Si flux supply.

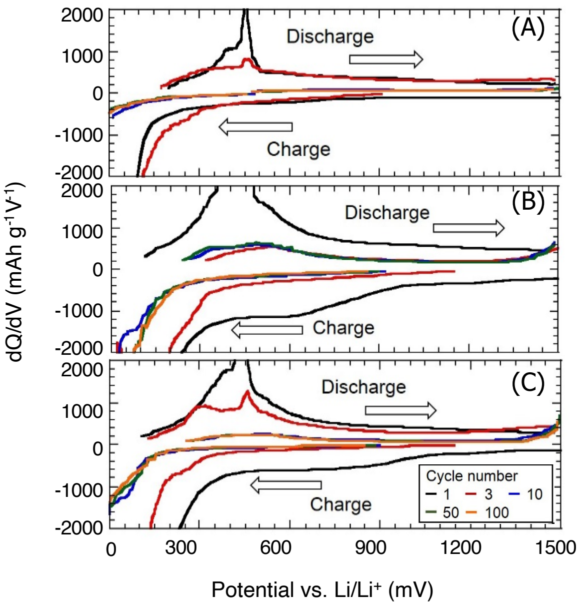

Figure S4. Differential capacity change dQ/dV at 1, 3, 10, 50 and 100th cycle of sPFE for condition I (A), I (B) and III (C).
